# Ingestion of Illicit Substances by Young Children Before and During the COVID-19 Pandemic

**DOI:** 10.1001/jamanetworkopen.2023.9549

**Published:** 2023-04-21

**Authors:** Brittany J. Raffa, Samantha Schilling, M. Katherine Henry, Victor Ritter, Colleen E. Bennett, Jeannie S. Huang, Natalie Laub

**Affiliations:** 1 Division of General Pediatrics and Adolescent Medicine, Department of Pediatrics, University of North Carolina at Chapel Hill; 2 The Cecil G. Sheps Center for Health Services Research, University of North Carolina at Chapel Hill; 3 Safe Place: Center for Child Protection and Health, Division of General Pediatrics, Children’s Hospital of Philadelphia, Philadelphia, Pennsylvania; 4 Department of Pediatrics, Perelman School of Medicine at the University of Pennsylvania, Philadelphia; 5 Clinical Futures, Children’s Hospital of Philadelphia, Philadelphia, Pennsylvania; 6 Division of Child Abuse Pediatrics, Department of Pediatrics, University of California at San Diego; 7 Department of Pediatrics, University of California San Diego

## Abstract

**Importance:**

Information about the trend in illicit substance ingestions among young children during the pandemic is limited.

**Objectives:**

To assess immediate and sustained changes in overall illicit substance ingestion rates among children younger than 6 years before and during the COVID-19 pandemic and to examine changes by substance type (amphetamines, benzodiazepines, cannabis, cocaine, ethanol, and opioids) while controlling for differing statewide medicinal and recreational cannabis legalization policies.

**Design, Setting, and Participants:**

Retrospective cross-sectional study using an interrupted time series at 46 tertiary care children’s hospitals within the Pediatric Health Information System (PHIS). Participants were children younger than 6 years who presented to a PHIS hospital for an illicit substance(s) ingestion between January 1, 2017, and December 31, 2021. Data were analyzed in February 2023.

**Exposure:**

Absence or presence of the COVID-19 pandemic.

**Main Outcome(s) and Measure(s):**

The primary outcome was the monthly rate of encounters for illicit substance ingestions among children younger than 6 years defined by *International Statistical Classification of Diseases, Tenth Revision, Clinical Modification* diagnosis code(s) for poisoning by amphetamines, benzodiazepines, cannabis, cocaine, ethanol, and opioids. The secondary outcomes were the monthly rate of encounters for individual substances.

**Results:**

Among 7659 children presenting with ingestions, the mean (SD) age was 2.2 (1.3) years and 5825 (76.0%) were Medicaid insured/self-pay. There was a 25.6% (95% CI, 13.2%-39.4%) immediate increase in overall ingestions at the onset of the pandemic compared with the prepandemic period, which was attributed to cannabis, opioid, and ethanol ingestions. There was a 1.8% (95% CI, 1.1%-2.4%) sustained monthly relative increase compared with prepandemic trends in overall ingestions which was due to opioids. There was no association between medicinal or recreational cannabis legalization and the rate of cannabis ingestion encounters.

**Conclusions and Relevance:**

In this study of illicit substance ingestions in young children before and during the COVID-19 pandemic, there was an immediate and sustained increase in illicit substance ingestions during the pandemic. Additional studies are needed to contextualize these findings in the setting of pandemic-related stress and to identify interventions to prevent ingestions in face of such stress, such as improved parental mental health and substance treatment services, accessible childcare, and increased substance storage education.

## Key Points

**Question:**

How have ingestion rates of illicit substances in young children changed before and during the COVID-19 pandemic?

**Findings:**

This cross-sectional study used an interrupted time series including 7659 children presenting with ingestions. There was an immediate increase in overall ingestions among children younger than 6 years in the pandemic period compared with the prepandemic period, attributed to cannabis, opioid, and ethanol ingestions, and there was a sustained monthly increase in overall ingestions attributed to opioids.

**Meaning:**

Additional research is needed to understand the reason for this study’s observed increased ingestion rate and to provide interventions and policies to address this trend.

## Teaser

This cross-sectional study evaluates the ingestion of illicit substances by young children before and during the COVID-19 pandemic.

## Introduction

The COVID-19 pandemic resulted in unprecedented challenges to work and family responsibilities; in the US, within the first year of the pandemic, more than two-thirds of childcare centers closed, more than 90% of schools transitioned to virtual learning, more than one-third of adults began working from home, and an additional 18 million adults became unemployed.^1,2,3,4,5^ Disruptions in substance use treatment centers and mental health care in general increased the risk of substance use,^6,7^ and a historic high in adult overdose deaths was reported.^8,9^ Intentional ingestions and ingestions occurring at home among youth younger than 20 years also increased during the pandemic.^8,9^ Among pediatric ingestions, children younger than 6 years compose the majority and more than 99% are unintentional.^10^ The presence of illicit substances in the home is particularly dangerous to young children, leading to potentially life-threatening outcomes when ingested. Although literature suggests the COVID-19 pandemic impacted adolescent and adult substance ingestions, to our knowledge, there is no literature evaluating rates of ingestions of illicit substances in young children before and during the pandemic.

Our objective was to examine trends in ingestion rates of illicit substances in young children before and during the COVID-19 pandemic. We included amphetamines, benzodiazepines, cannabis, cocaine, ethanol, and opioids as illicit substances. Although many of these substances are not illegal, their nonmedical use among young children is illegal and/or illicit. We hypothesized that there would be an increase in illicit substance ingestions during the pandemic period. We also hypothesized that cannabis would compose the majority of ingestions and that there would be an increase in the cannabis ingestion rate due to its relative ease of access, perceived innocuous effects, and increase in medicinal and recreational cannabis legalization.

## Methods

### Study population and setting

Data for this retrospective cross-sectional study were obtained from the Pediatric Health Information System (PHIS), a comparative database with clinical and resource use data for emergency department (ED), observation unit, and inpatient encounters from 49 tertiary care children’s hospitals.^11^ Included data are deidentified and subjected to rigorous reliability and validity checks.^12^ During the study period, 46 of the hospitals contributed full data and were included. This study was reviewed and determined exempt by the University of California San Diego institutional review board, who granted a waiver of informed consent as the research was deemed minimal risk, did not adversely affect the rights and welfare of the participants, and could not practicably carried out without the waiver. The Strengthening the Reporting of Observational Studies in Epidemiology (STROBE) reporting guideline on cross-sectional studies was followed.^13,14^

Children younger than 6 years with an ED, observational, or inpatient encounter at hospitals contributing data to PHIS with *International Statistical Classification of Diseases, Tenth Revision, Clinical Modification (ICD-10-CM) *diagnosis code(s) for ingestions of amphetamines, benzodiazepines, cannabis, cocaine, ethanol, or opioids between January 1, 2017, and December 31, 2021 were included (eTable in Supplement 1). Birth hospitalization encounters were excluded. Encounters were unique at the patient-substance-month level (ie, ED encounters and observation unit or inpatient encounters were not double counted but a patient who ingested 2 substances in 1 encounter was counted twice; patients presenting for an ingestion at 2 points in time were counted for each encounter).

For all encounters meeting inclusion criteria, the following data were abstracted: patient demographics (age, sex, and insurance), all *ICD-10-CM* codes associated with the encounter, encounter type (ED, observation, inpatient), hospital identification, encounter dates, and intensive care unit (ICU) status. We also obtained the number of all-cause ED, observation, and inpatient encounters for the study period per hospital per year.

### Measures and Outcomes

The primary outcome was the monthly rate of encounters for illicit substance ingestions among children younger than 6 years. The secondary outcomes were the monthly rate of encounters for individual substance ingestion categories (amphetamines, benzodiazepines, cannabis, cocaine, ethanol, and opioids) among children younger than 6 years. The outcome encounter rate is defined as the number of ingestions per month per 10 000 all-cause hospital encounters calculated using the rolling 12-month mean for all-cause hospital encounters to account for seasonal fluctuations in number of encounters. Because hospital encounters substantially decreased during the first several months of the pandemic, which would falsely elevate the illicit substance ingestion rate, the total encounters for 2019 were used for the denominator for rates in 2020 and 2021.^15,16,17,18,19^

The primary variables were time in months and the absence or presence of the pandemic with the pandemic period starting on April 1, 2020. Ingestion encounters from March 2020 were excluded from analyses as shelter-in-place orders, physical distancing restrictions, and daycare and school closures were implemented at different times across PHIS hospital catchment areas from March 10, 2020, to March 31, 2020.

For the outcomes of all illicit substance ingestion encounters and cannabis ingestion encounters, the models included 2 additional independent variables for medicinal and recreational cannabis legalization status. For each hospital month, the medicinal and recreational cannabis legalization variables were binary (absent or present). For each hospital located in a state where cannabis legalization started at some point during the study period, legalization was coded as present the month following the date in which the state passed or enacted legislation legalizing recreational cannabis use (eg, if legislation for recreational cannabis was passed January 15, 2019, recreational legalization would be coded as present starting in February 2019). In 11 states and the District of Columbia (covering 26 hospitals in our sample) medicinal cannabis was already legal at the start of the study; 4 additional states (4 hospitals) legalized medicinal cannabis during the study period. Three states and the District of Columbia (10 hospitals) legalized recreational cannabis before the study period, and 4 states (7 hospitals) legalized recreational cannabis during the study period.

### Statistical Analysis

Descriptive statistics were used to describe patients and encounters with illicit substance ingestions before and during the pandemic using SAS version 9.4 (SAS Institute) and R version 4.1.2 (R Project for Statistical Computing). All *P*-values reported are 2-sided, and a significance level of 5% was assumed. For the primary analysis, we examined if the pandemic was associated with the rate of illicit ingestion encounters. For the 59-month study period, we performed an interrupted time series (ITS) analysis using negative binomial regression models fitted via generalized estimating equations accounting for clustering by hospital.^20^ A first-order autoregressive working correlation structure was used. Our exposure was time reflected by the month of interest, with 39 months before the pandemic (January 2017 to February 2020) and 20 months following the pandemic onset (April 2020 to December 2021). Medicinal and recreational cannabis legalization statuses were included as independent variables.

For the secondary analysis, we stratified by substance type and examined if the COVID-19 pandemic was associated with the rate of ingestion-related encounters for the following substances: amphetamines, benzodiazepines, cannabis, cocaine, ethanol, and opioids. The ITS analysis described previously was repeated 6 times, using each individual substance category as the outcome. The cannabis model included medicinal and recreational cannabis legalization status variables.

For all ITS models, we assessed the prepandemic baseline trends as well as the immediate and sustained monthly outcomes of the pandemic on illicit ingestion rates. We calculated projected ingestion rates for the pandemic period as if the pandemic never occurred. This hypothetical scenario was compared with the observed ingestion rates to examine whether actual rates differed from expected values. Thus, for the combined ingestion model and the individual ingestion models, we present an immediate change in the encounter rate for ingestions in children younger than 6 years attributed to the pandemic between February 29, 2020, and April 1, 2020, (eg, there was a 50% immediate increase in ingestion rate following the onset of the pandemic) and a monthly change in the ingestion rate thereafter (eg, starting April 1, 2020, in addition to the baseline trend, each month the ingestion rate increased by 3%).

The ITS model is calculated as follows: log(μ*_ij_*/*n_ij_)* = β_0_ + β*_1_time_j_* + β*_2_pos_j _*+ β*_3_time_pos_j,_* where μ*_ij_* represents the expected number of ingestions for hospital *i* at time *j* (in months), *n_ij _*represents the total number of encounters for hospital *i* at time *j*, β_0 _represents the baseline expected number of ingestions (at time = 0, prepandemic and no cannabis legalization), β*_1_* represents the baseline monthly trend, β*_2_* represents the immediate level change following the pandemic, β*_3_* represents the sustained monthly outcome, and *pos_j_* corresponds to a dummy variable indicating the prepandemic period (0) or the pandemic period (1). Additional coefficients (β_4_ and β_5_) representing medicinal and recreational cannabis legalization were included in the overall and cannabis ingestion models.

## Results

Among 7659 children presenting with ingestions, the mean (SD) age was 2.2 (1.3) years. More than half (5825 participants [76.0%]) of children were Medicaid insured or self-pay, 1672 (22.0%) were privately insured, and 162 (2.1)% were missing/other payer (Table 1). The percentage of encounters requiring an ICU stay remained consistent before and during the pandemic at 18%. The analysis was conducted at the substance encounter level (7758) and in total there were 3809 substance encounters in the prepandemic period and 3850 substance encounters in the pandemic period. There were 214 encounters in which the patient ingested 2 substances and were therefore counted twice in the model. There were 7 encounters in which the patient ingested 3 substances and were therefore counted 3 times, and 99 patients presented for 2 separate ingestion encounters during the study period.

**Table 1.** Study Population and Illicit Substance Type

Characterics	No. (%)		
	Prepandemic period (January 1, 2017, to February 29, 2020) (n = 3809)	Pandemic period (April 1, 2020, to December 31, 2021) (n = 3850)	Total period (January 1, 2017, to December 31, 2021) (N = 7659)
Sex			
Female	1829 (48)	1916 (50)	3745 (49)
Male	1978 (52)	1927 (50)	3905 (51)
Missing	2	7	9
Age, y			
0-1	2049 (54)	1904 (49)	3953 (52)
2-3	1352 (35)	1395 (36)	2747 (36)
4-5	408 (11)	551 (14)	959 (13)
Insurance^a^			
Medicaid or self-pay	2844 (75)	2981 (77)	5825 (76)
Private	905 (24)	767 (20)	1672 (22)
Missing or other	60 (1.6)	102 (2.6)	162 (2.1)
Encounters, No.	3856	3902	7758
Encounter type			
ED	1342 (35)	1110 (28)	2452 (32)
Observation	963 (25)	1318 (34)	2281 (29)
Inpatient	1551 (40)	1474 (38)	3025 (39)
ICU stay	690 (18)	710 (18)	1400 (18)
Ingestion^c^			
Amphetamine	1119 (29)	720 (18)	1839 (24)
Benzodiazepine	764 (20)	309 (7.9)	1073 (14)
Cannabis	926 (24)	1900 (49)	2826 (36)
Cocaine	184 (4.8)	171 (4.4)	355 (4.6)
Ethanol	153 (4.0)	162 (4.2)	315 (4.1)
Opioid	803 (21)	775 (20)	1578 (20)
Polysubstances	91 (2.4)	130 (3.3)	221 (2.8)

Abbreviations: ED, emergency department; ICU, intensive care unit.

^a^ Medicaid or self-pay includes children on Medicaid, Children’s Health Insurance Program, or self-pay. Private insurance includes children on any commercial insurance or Tricare. Missing includes all children with missing or “other payer.”

^c^ As described in the Results section, some participants ingested multiple substances.

### Interrupted Time Series Analysis

**Figure 1.**
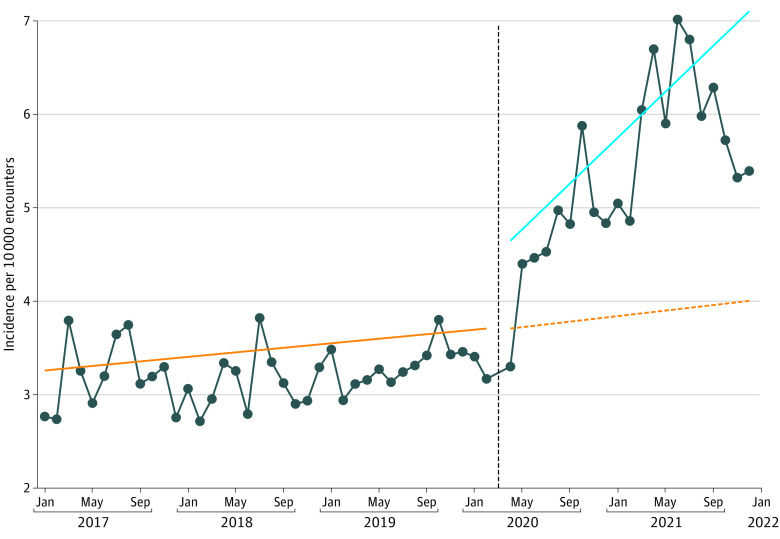
Illicit Ingestion Incidence Rate Among Children Younger Than 6 Years at Pediatric Health Information System Hospitals Interrupted time series results of the number of illicit (cannabis, opioids, benzodiazepines, cocaine, ethanol, and amphetamines) ingestions per month per 10 000 all cause hospital encounters before and during the COVID-19 pandemic. Medicinal and recreational cannabis legalization laws were included as covariates. The study period includes 39 months in the prepandemic period (January 2017 to February 2020) and 20 months in the pandemic period (April 2020 to December 2021). Ingestion encounters from March 2020 were excluded. The solid orange line depicts the prepandemic ingestion rate trend. The vertical dashed black line depicts the onset of the pandemic. The dashed orange line depicts the projected ingestion rate according to the prepandemic trend had the pandemic not occurred. The solid blue line depicts the pandemic period trend.

We observed a 25.6% (95% CI, 13.2%-39.4%; *P* < .001) immediate increase in the overall ingestion rate among encounters at the onset of the pandemic compared with the prepandemic period, followed by a 1.8% (95% CI, 1.1%-2.4%; *P* < .001) sustained monthly increase in the overall ingestion rate per month (Table 2 and Figure 1). Medicinal and recreational cannabis legalization laws were not associated with significant changes in the overall ingestion rates (Table 2).

**Table 2.** Interrupted Time Series Results^a^

Drug group	Parameter^b^	IRR (95% CI)	*P* value
Overall	Intercept	3.199 (2.697-3.794)	<.001
	Time	1.003 (1.000-1.007)	.06
	Sustained change	1.018 (1.011-1.024)	<.001
	Immediate change	1.256 (1.132-1.394)	<.001
	Recreational THC	0.868 (0.642-1.173)	.36
	Medicinal THC	1.043 (0.789-1.380)	.76
Cannabis	Intercept	0.468 (0.330-0.664)	<.001
	Time	1.022 (1.014-1.029)	<.001
	Sustained change	1.006 (0.994-1.018)	.34
	Immediate change	1.707 (1.485-1.961)	<.001
	Recreational THC	1.294 (0.950-1.762)	.10
	Medicinal THC	1.060 (0.734-1.530)	.76
Opioids	Intercept	0.834 (0.684-1.015)	.07
	Time	0.991 (0.985-0.997)	.004
	Sustained change	1.049 (1.034-1.064)	<.001
	Immediate change	1.275 (1.040-1.562)	.02
Benzodiazepines	Intercept	0.855 (0.698-1.046)	.13
	Time	0.985 (0.978-0.993)	<.001
	Sustained change	1.008 (0.987-1.030)	.46
	Immediate change	0.954 (0.730-1.246)	.73
Cocaine	Intercept	0.135 (0.078-0.235)	<.001
	Time	1.012 (1.000-1.024)	.04
	Sustained change	1.024 (0.995-1.054)	.11
	Immediate change	0.827 (0.558-1.224)	.34
Ethanol	Intercept	0.138 (0.093-0.203)	<.001
	Time	0.997 (0.980-1.014)	.73
	Sustained change	1.006 (0.974-1.039)	.71
	Immediate change	1.808 (1.037-3.151)	.04
Amphetamines	Intercept	0.865 (0.729-1.027)	.10
	Time	1.005 (1.000-1.011)	.06
	Sustained change	0.989 (0.978-1.000)	.05
	Immediate change	1.021 (0.871-1.197)	.80

Abbreviation: IRR, incidence rate ratio; THC, tetrahydrocannabinol.

^a^ Illicit for medicinal and recreational cannabis legalization laws in ITS model. Of note, states have diverse laws on edibles but the majority with recreational cannabis legalizations laws permit edibles.

^b^ Intercept represents the baseline level of the outcome; time represents the monthly baseline rate of change; recreational THC indicates the presence or absence of recreational marijuana legalization; medicinal THC indicates the presence or absence of medicinal marijuana legalization.

**Figure 2.**
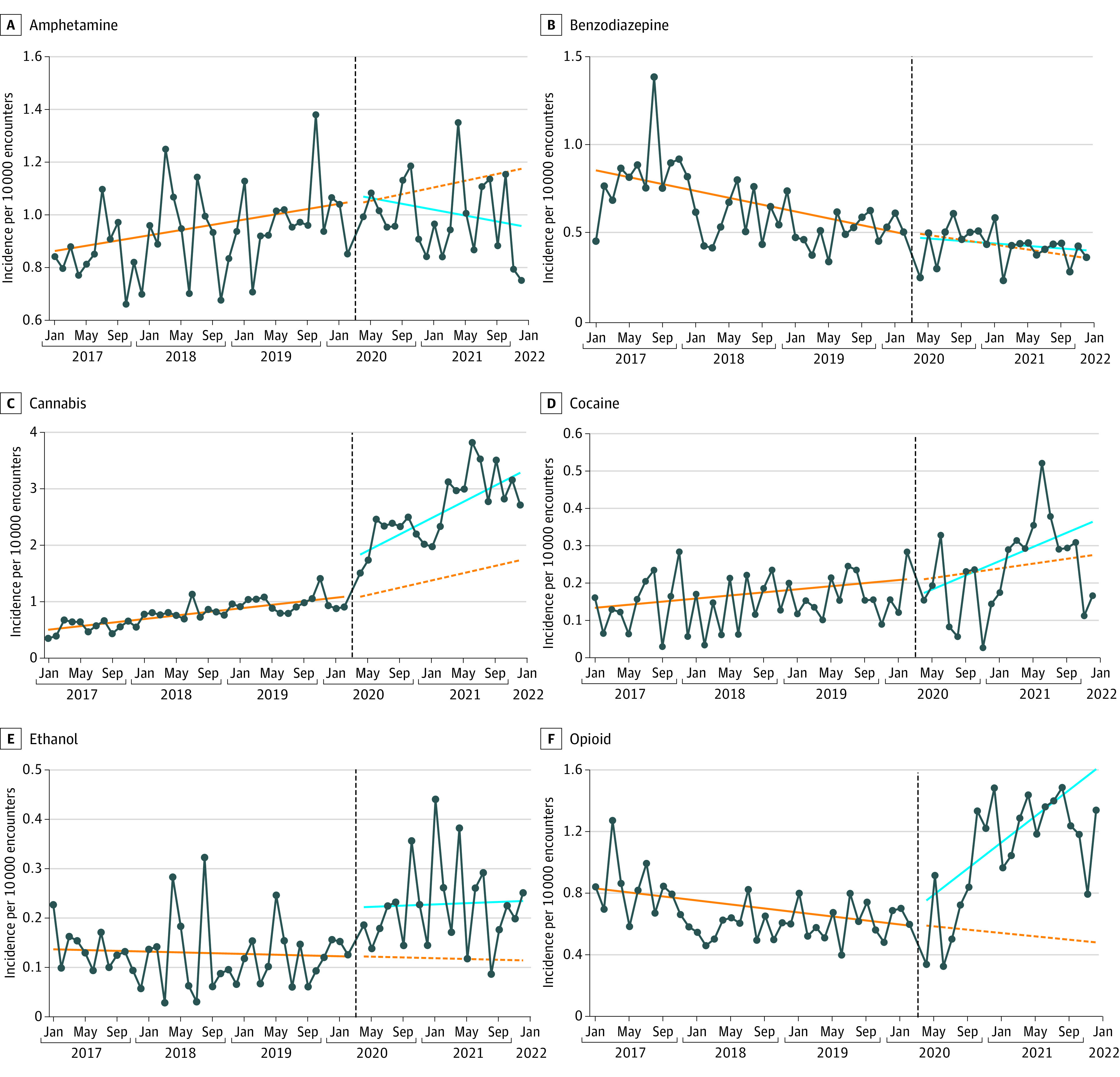
Ingestion Incidence Rate by Substance Type Please note because of large differences in magnitude for difference substances, scales are different on each panel. The solid orange line depicts the prepandemic ingestion rate trend. The vertical dashed black line depicts the onset of the pandemic. The dashed orange line depicts the projected ingestion rate according to the prepandemic trend had the pandemic not occurred. The solid blue line depicts the pandemic period trend.

When controlling for medicinal and recreational cannabis legalization laws, there was a 70.7% (95% CI, 48.5%-96.1%; *P* < .001) immediate increase in cannabis ingestion encounters at the onset of the pandemic. After this immediate observed increase, there was no additional significant monthly increase in cannabis ingestions in the pandemic period above the baseline trend. Of note, in the prepandemic period, cannabis ingestions were increasing at a rate of 2.2% (95% CI, 1.4%-2.9%, *P* < .001) per month. After the immediate increase following the onset of the pandemic, this monthly increase continued but did not change significantly (Table 2, Figure 2). In the cannabis model, neither medicinal cannabis legalization nor recreational cannabis legalization were associated with change in the rate of cannabis ingestion encounters (Table 2).

There was no significant immediate increase in individual substance ingestion rates for amphetamines, cocaine, or benzodiazepines at the onset of the pandemic (Table 2 and Figure 2). There was a 27.5% (95% CI, 4.0%-56.2%; *P* = .02) immediate increase in opioid ingestions among encounters at the onset of the pandemic compared with the prepandemic period. There was an 80.8% (95% CI, 3.7%-215.1%; *P* = .04) immediate increase in ethanol ingestions among encounters at the onset of the pandemic compared with the prepandemic period.

There was a significant sustained monthly increase in the individual ingestion rate for opioids (4.9% increase per month; 95% CI, 3.4% to 6.4%; *P* < .001) and decrease in amphetamines (1.1% decrease per month; 95% CI, −2.2% to 0%; *P* = .05) during the pandemic period (Table 2 and Figure 2). There were no significant sustained monthly changes in the individual ingestion rates for benzodiazepines, cocaine, and ethanol.

According to the modeling, we observed the following cumulative increase in ingestions in addition to the baseline projected trend since the beginning of the pandemic (April 2020) until December 2021: 40.9 more overall ingestions per 10 000 encounters, 23.9 more ingestions of cannabis per 10 000 encounters, 12.4 more ingestions of opioids per 10 000 encounters, and 2.3 more ingestions of ethanol per 10 000 encounters.

## Discussion

Our study found an immediate and sustained monthly increase in the rate of hospital encounters for illicit substance ingestions among young children during the COVID-19 pandemic compared with the prepandemic period. Cannabis, opioids, and ethanol were the substances associated with an immediate increase in ingestion encounters. Opioids were associated with a sustained monthly increase in illicit substance encounters during the pandemic period. Neither medicinal nor recreational legalization of cannabis was statistically associated with an increase in the rate of cannabis ingestion encounters among hospitals located in states in which cannabis was legalized.

This is the first study to our knowledge to examine ingestions of illicit substances during the COVID-19 pandemic among young children. Although prior studies identified that, during the early pandemic, there was an increase in adolescent ingestions and ingestions occurring at home, despite an overall decrease in ingestions among children 19 years and younger, our study addresses a gap in the literature by specifically examining illicit substance ingestions among young children and by including the associations between cannabis medicinal and recreational legalization policy and observed ingestion rates.^9,21^ Furthermore, our study uses administrative PHIS data, which have higher accuracy and reliability compared with poison control center data used in prior studies.

Although our study does not explore mechanisms that led to the observed increased ingestion rate among young children, other studies^6,22,23,24,25,26,27,28^ have described the association of the pandemic with increased stress, worsened mental health, increased substance use among parents, and disruption of substance use treatment. For instance, 13% of adults reported initiating or increasing substance use to cope with pandemic-related stress, and 27% of parents reported worsening mental health for themselves following the pandemic, which was worse among parents with younger children compared with those with older children.^29,30^ Furthermore, although many families experienced loss of childcare, this was more common among those with children younger than 5 years in the home.^29^ This combination of increased illicit substance use by caregivers and increased exposure to substances in the home due to childcare closures may be associated with the observed increase in illicit substance ingestion among young children during the pandemic. Inadequate substance safekeeping may also be a factor as less than half of surveyed parents in a state where cannabis is legal recreationally and medicinally report safe storage.^31^

The immediate and sustained increase in opioid ingestions observed in our study occurred during the largest ever increase in adult overdose deaths, largely associated with synthetic opioids.^28,32,33,34^ The observed increase in polysubstance ingestions among young children during the pandemic is consistent with the increase in polysubstance ingestions among adults during the pandemic.^35,36^ Interestingly, we did not observe an increase in severity of presentations in the form of ICU admissions, despite an increase in ingestions. Although we examined severity of all combined substances among young children, this observation differs from the increase in severe intoxications from cannabis among children aged 0 to 18 years following the recreational cannabis legalization in Canada.^37^

In our analysis, the onset of the pandemic was associated with a large immediate increase in cannabis ingestions. We did not identify a statistically significant increase in cannabis ingestions in states following medicinal and recreational legalization. Prior analysis of the impact of cannabis legalization on hospital encounters for pediatric ingestions has shown mixed results: no significant change in encounters following legalization of recreational cannabis in Canada, but an increase in the severity of encounters^37^; an increase in the cannabis ingestion rate following legalization of recreational cannabis in Colorado, with edible products responsible for more than half of encounters^38^; and no effect of state recreational or medical legalization policies on pediatric cannabis ingestions despite a more than 13-fold increase in the ingestion rate between 2004 and 2018.^39^

The increased sample size of our study, interrupted time series method, and the increasing number of states enacting cannabis legalization policies since 2018 when the last major analysis occurred (between 2019 to 2021, 2 states legalized medicinal cannabis and 6 states legalized recreational cannabis) are all strengths of our study.^39^ These findings provide important information on the impact of state medicinal and recreational cannabis legalization policy on illicit substance ingestions in young children, for which previous studies have shown contradictory results.^37,38,39^

### Limitations

Our study has several important limitations. First, although PHIS includes hospitals located in multiple states, this does not constitute a nationally representative sample, thereby limiting generalizability. There is also the risk of inaccurate *ICD-10-CM* documentation; however, we believe this would underestimate the observed rates and would likely be consistent over the study period. Our model accounts for cannabis policy; however, the date that legislation is passed may not be the date the policy goes into effect. Although our reported ingestion rates may include ingestion of cannabis prescribed to the child by a physician, we believe that, even in states that have legalized medicinal cannabis, it is rarely prescribed to children younger than 6 years. On the other hand, it is likely that children in this age range may accidently ingest cannabis prescribed by physicians to adults due to suboptimal storage and/or supervision. Another limitation includes using the word illicit to describe substances that may have been medically prescribed; however, the nonmedical use of these substances among young children is illegal and often results in a report to child protective services. Additionally, with substance use increasing in the general public, pediatric health care clinicians may now be ordering more drug screen tests among children compared with historical ordering patterns and therefore surveillance bias is a possible limitation.

## Conclusions

This study found an immediate and sustained monthly increase in illicit substance ingestions among young children during the pandemic, attributable to multiple illicit substances. Our study also suggested that medicinal and recreational cannabis legalization policies are not statistically associated with increased cannabis ingestions among children younger than 6 years. These findings suggest the need for interventions to address factors that may be associated with the observed increase in illicit substance ingestions. Such interventions may include supporting policies that buffer family stress, improving parent access to and affordability of mental health and substance treatment services, increasing availability of childcare, and providing education about safe storage of substances in the home. Although childcare centers and schools have now reopened, travel has resumed, and many describe a postpandemic period in sight, the new normal will not be a return to prepandemic norms. Hybrid work schedules, health care workforce shortages, insufficient mental health and substance use centers, and costly and inaccessible childcare options will continue to be a reality that must be addressed to counter illicit substance ingestions among young children.

## Notes

Supplement 1. **eTable. ***ICD-10* Diagnoses

Supplement 2.
Data Sharing Statement

## References

[1] Lee EK, Parolin Z. The care burden during COVID-19: a national database of child care closures in the United States. Socius: Sociol Res Dyn World. 2021;7. doi:10.1177/23780231211032028

[2] Teleworking and lost work during the pandemic: new evidence from the CPS. US Bureau of Labor Statistics. 2021. Accessed June 13, 2022. https://www.bls.gov/opub/mlr/2021/article/teleworking-and-lost-work-during-the-pandemic-new-evidence-from-the-cps.htm

[3] Igielnik R. A rising share of working parents in the U.S. say it’s been difficult to handle child care during the pandemic. Pew Research Center. January 26, 2021. Accessed May 30, 2022. https://www.pewresearch.org/fact-tank/2021/01/26/a-rising-share-of-working-parents-in-the-u-s-say-its-been-difficult-to-handle-child-care-during-the-pandemic/

[4] McElrath K. Nearly 93% of households with school-age children report some form of distance learning during COVID-19. United States Census Bureau. August 26, 2020. Accessed August 8, 2022. https://www.census.gov/library/stories/2020/08/schooling-during-the-covid-19-pandemic.html

[5] Labor force statistics from the current population survey. US Bureau of Labor Statistics. Accessed August 8, 2022. https://www.bls.gov/cps/effects-of-the-coronavirus-covid-19-pandemic.htm

[6] Joudrey PJ, Adams ZM, Bach P, . Methadone access for opioid use disorder during the COVID-19 pandemic within the United States and Canada. JAMA Netw Open. 2021;4(7):e2118223. doi:10.1001/jamanetworkopen.2021.18223 34297070 PMC8303098

[7] The impact of COVID-19 on mental, neurological and substance use services: results of a rapid assessment. World Health Organization. October 5, 2020. Accessed March 15, 2023. https://apps.who.int/iris/rest/bitstreams/1310579/retrieve

[8] Drug overdose deaths in the U.S. up 30% in 2020. Centers for Disease Control and Prevention, National Center for Health Statistics; July 14, 2021. Accessed March 15, 2023. https://www.cdc.gov/nchs/pressroom/nchs_press_releases/2021/20210714.htm

[9] Lelak KA, Vohra V, Neuman MI, Farooqi A, Toce MS, Sethuraman U. COVID-19 and pediatric ingestions. Pediatrics. 2021;148(1):e2021051001. doi:10.1542/peds.2021-051001 33906928

[10] Poison statistics: national data 2020. Poison Control National Capital Poison Center. 2022. Accessed June 14, 2022. https://www.poison.org/poison-statistics-national

[11] Leverage clinical and resource utilization data. Children's Hospital Association. Accessed August 13, 2022, https://www.childrenshospitals.org/content/analytics/product-program/pediatric-health-information-system?

[12] Fletcher DM. Achieving data quality. How data from a pediatric health information system earns the trust of its users. J AHIMA. 2004;75(10):22-26. 15559835

[13] The Strengthening the Reporting of Observational Studies in Epidemiology (STROBE) Statement: guidelines for reporting observational studies. 2022. Accessed March 15, 2023. https://www.equator-network.org/reporting-guidelines/strobe/10.1136/bmj.39335.541782.AD PMC2034723 17947786

[14] von Elm E, Altman DG, Egger M, Pocock SJ, Gøtzsche PC, Vandenbroucke JP; STROBE Initiative. The Strengthening the Reporting of Observational Studies in Epidemiology (STROBE) statement: guidelines for reporting observational studies. Lancet. 2007;370(9596):1453-1457. doi:10.1016/S0140-6736(07)61602-X 18064739

[15] Jeffery MM, D’Onofrio G, Paek H, . Trends in emergency department visits and hospital admissions in health care systems in 5 states in the first months of the COVID-19 pandemic in the US. JAMA Intern Med. 2020;180(10):1328-1333. doi:10.1001/jamainternmed.2020.3288 32744612 PMC7400214

[16] Hartnett KP, Kite-Powell A, Devies J, . Impact of the COVID-19 pandemic on emergency department visits — United States, January 1, 2019–May 30, 2020. MMWR Morb Mortal Wkly Rep. 2020;69(23):699-704. doi:10.15585/mmwr.mm6923e1 32525856 PMC7315789

[17] Symum H, Zayas-Castro J. Impact of the COVID-19 pandemic on the pediatric hospital visits: evidence from the state of Florida. Pediatric Reports. 2022;14(1):58-70. doi:10.3390/pediatric14010010 35225879 PMC8883905

[18] Pelletier JH, Rakkar J, Au AK, Fuhrman D, Clark RSB, Horvat CM. Trends in US pediatric hospital admissions in 2020 compared with the decade before the COVID-19 pandemic. JAMA Netw Open. 2021;4(2):e2037227. doi:10.1001/jamanetworkopen.2020.37227 33576819 PMC7881361

[19] Radhakrishnan L, Carey K, Hartnett KP, . Pediatric emergency department visits before and during the COVID-19 pandemic: United States, January 2019–January 2022. MMWR Morb Mortal Wkly Rep. 2022;71(8):313-318. doi:10.15585/mmwr.mm7108e1 35202351

[20] Schaffer AL, Dobbins TA, Pearson S-A. Interrupted time series analysis using autoregressive integrated moving average (ARIMA) models: a guide for evaluating large-scale health interventions. BMC Med Res Methodol. 2021;21(1):58. doi:10.1186/s12874-021-01235-8 33752604 PMC7986567

[21] Schultz BRE, Hoffmann JA, Clancy C, Ramgopal S. Hospital encounters for pediatric ingestions before and during the COVID-19 pandemic. Clin Toxicol (Phila). 2022;60(2):269-271. doi:10.1080/15563650.2021.1925687 33988077

[22] Stress in America. One year later, a new wave of pandemic health concerns. 2021. Accessed March 15, 2023. https://www.apa.org/news/press/releases/stress/2021/sia-pandemic-report.pdf

[23] Our COVID-19 pulse survey data. HRSA Maternal and Child Health Bureau. 2021. Accessed March 15, 2023. https://mchb.hrsa.gov/covid-19/data

[24] Harry EM, Carlasare LE, Sinsky CA, . Childcare stress, burnout, and intent to reduce hours or leave the job during the COVID-19 pandemic among US health care workers. JAMA Netw Open. 2022;5(7):e2221776. doi:10.1001/jamanetworkopen.2022.21776 35849398 PMC9294994

[25] Kotlar B, Gerson E, Petrillo S, Langer A, Tiemeier H. The impact of the COVID-19 pandemic on maternal and perinatal health: a scoping review. Reprod Health. 2021;18(1). doi:10.1186/s12978-021-01070-6 PMC7812564 33461593

[26] Dodge KA, Skinner AT, Godwin J, . Impact of the COVID-19 pandemic on substance use among adults without children, parents, and adolescents. Addict Behav Rep. 2021;14:100388. doi:10.1016/j.abrep.2021.100388 34938846 PMC8664966

[27] Moreland-Russell S, Jabbari J, Ferris D, Roll S. At home and on the brink: U.S. parents’ mental health during COVID-19. Int J Environ Res Public Health. 2022;19(9):5586. doi:10.3390/ijerph19095586 35564980 PMC9102023

[28] Slavova S, Rock P, Bush HM, Quesinberry D, Walsh SL. Signal of increased opioid overdose during COVID-19 from emergency medical services data. Drug Alcohol Depend. 2020;214:108176. doi:10.1016/j.drugalcdep.2020.108176 32717504 PMC7351024

[29] Patrick SW, Henkhaus LE, Zickafoose JS, . Well-being of parents and children during the COVID-19 pandemic: a national survey. Pediatrics. 2020;146(4):e2020016824. doi:10.1542/peds.2020-016824 32709738

[30] Czeisler MÉ, Lane RI, Petrosky E, . Mental health, substance use, and suicidal ideation during the COVID-19 pandemic - United States, June 24-30, 2020. MMWR Morb Mortal Wkly Rep. 2020;69(32):1049-1057. doi:10.15585/mmwr.mm6932a1 32790653 PMC7440121

[31] Gimelli A, Deshpande A, Magana J, Moulin A. Cannabis in homes with children: a survey on use, storage, and attitudes. West J Emerg Med. 2021;22(5):1146-1149. doi:10.5811/westjem.2021.5.49057 34546891 PMC8463057

[32] A time of crisis for the opioid epidemic in the USA. The Lancet. 2021;398(10297):277. doi:10.1016/S0140-6736(21)01653-6 PMC8482059 34303422

[33] Drug overdose deaths in the U.S. top 100,000 annually. National Center for Health Statistics. 2021. Accessed March 15, 2023. https://www.cdc.gov/nchs/pressroom/nchs_press_releases/2021/20211117.htm

[34] Trends and statistics overdose death rates. National Institute on Drug Abuse. Accessed January 20, 2023, https://nida.nih.gov/research-topics/trends-statistics/overdose-death-rates

[35] O’Donnell J, Tanz LJ, Gladden RM, Davis NL, Bitting J. Trends in and characteristics of drug overdose deaths involving illicitly manufactured fentanyls: United States, 2019-2020. MMWR Morb Mortal Wkly Rep. 2021;70(50):1740-1746. doi:10.15585/mmwr.mm7050e3 34914673 PMC8675656

[36] Vignes K, Cockerham C, Su L, . Trends in maternal polysubstance use during the COVID-19 pandemic (poster). Am J Obstet Gynecol. 2022;226(1):S621-S622. doi:10.1016/j.ajog.2021.11.1023

[37] Cohen N, Galvis Blanco L, Davis A, . Pediatric cannabis intoxication trends in the pre and post-legalization era. Clin Toxicol (Phila). 2022;60(1):53-58. doi:10.1080/15563650.2021.1939881 34137352

[38] Wang GS, Le Lait M-C, Deakyne SJ, Bronstein AC, Bajaj L, Roosevelt G. Unintentional pediatric exposures to marijuana in Colorado, 2009-2015. JAMA Pediatr. 2016;170(9):e160971. doi:10.1001/jamapediatrics.2016.0971 27454910

[39] Bennett CE, Venkataramani A, Henretig FM, Faerber J, Song L, Wood JN. Recent trends in marijuana-related hospital encounters in young children. Acad Pediatr. 2022;22(4):592-597. doi:10.1016/j.acap.2021.07.018 34325061

