# Supplementary material for: Ingestion of Illicit Substances by Young Children Before and During the COVID-19 Pandemic
Source: JAMA Netw Open. 2023 Apr 21;6(4):e239549. doi: 10.1001/jamanetworkopen.2023.9549 (PMC10122182; doi:10.1001/jamanetworkopen.2023.9549)
Supplement: Supplement 1. — eTable. ICD-10 Diagnoses [file jamanetwopen-e239549-s001.pdf]

Supplemental Online Content

Raffa BJ, Schilling S, Henry MK, et al. Ingestion of illicit substances by young children before and during the COVID-19 pandemic. *JAMA Netw Open*. 2023;6(4):e239549.
doi:10.1001/jamanetworkopen.2023.9549

eTable. *ICD-10* Diagnoses

This supplemental material has been provided by the authors to give readers additional information about their work.

© 2023 Raffa BJ et al. *JAMA Network Open.*

eTable: *ICD-10* Diagnoses

ICD-10 Code:	Description:
F11929	opioid intoxication
F1192	opioid intoxication
F1095	Alcohol use/intoxication
F1095	Alcohol use/intoxication
F1098	Alcohol use/intoxication
F10159	Alcohol use/intoxication
F1090	Alcohol use, unspecified, uncomplicated
F1091	Alcohol use, unspecified, in remission
F1092	Alcohol use/intoxication
F10920	Alcohol use/intoxication
F109	Alcohol use/intoxication
F10921	Alcohol use/intoxication
F10929	Alcohol use/intoxication
F10930	Alcohol use, unspecified with withdrawal, uncomplicated
F10931	Alcohol use, unspecified with withdrawal delirium
F10932	Alcohol use, unspecified with withdrawal with perceptual disturbance
F10939	Alcohol use, unspecified with withdrawal, unspecified
F1094	Alcohol use, unspecified with alcohol-induced mood disorder
F10950	Alcohol use, unspecified with alcohol-induced psychotic disorder with delusions
F10951	Alcohol use, unspecified with alcohol-induced psychotic disorder with hallucinations
F10959	Alcohol use, unspecified with alcohol-induced psychotic disorder, unspecified
F1096	Alcohol use, unspecified with alcohol-induced persisting amnestic disorder
F1097	Alcohol use, unspecified with alcohol-induced persisting dementia
F10980	Alcohol use, unspecified with alcohol-induced anxiety disorder
F10981	Alcohol use, unspecified with alcohol-induced sexual dysfunction
F10982	Alcohol use, unspecified with alcohol-induced sleep disorder
F10988	Alcohol use, unspecified with other alcohol-induced disorder
F12	Cannabis use unspecified
F1099	Alcohol use, unspecified with unspecified alcohol-induced disorder
F1210	Cannabis abuse, uncomplicated
F1211	Cannabis abuse, in remission
F1212	Cannabis abuse with intoxication
F12120	Cannabis abuse with intoxication, uncomplicated
F12121	Cannabis abuse with intoxication delirium

© 2023 Raffa BJ et al. *JAMA Network Open.*

F12122	Cannabis abuse with intoxication with perceptual disturbance
F12129	Cannabis abuse with intoxication, unspecified
F1213	Cannabis abuse with withdrawal
F12150	Cannabis abuse with psychotic disorder with delusions
F12151	Cannabis abuse with psychotic disorder with hallucinations
F12159	Cannabis abuse with psychotic disorder, unspecified
F12180	Cannabis abuse with cannabis-induced anxiety disorder
F12188	Cannabis abuse with other cannabis-induced disorder
F1219	Cannabis abuse with unspecified cannabis-induced disorder
F1220	Cannabis dependence, uncomplicated
F1221	Cannabis dependence, in remission
F12220	Cannabis dependence with intoxication, uncomplicated
F12221	Cannabis dependence with intoxication delirium
F12222	Cannabis dependence with intoxication with perceptual disturbance
F12229	Cannabis dependence with intoxication, unspecified
F1223	Cannabis dependence with withdrawal
F12250	Cannabis dependence with psychotic disorder with delusions
F12251	Cannabis dependence with psychotic disorder with hallucinations
F12259	Cannabis dependence with psychotic disorder, unspecified
F12280	Cannabis dependence with cannabis-induced anxiety disorder
F12288	Cannabis dependence with other cannabis-induced disorder
F1229	Cannabis dependence with unspecified cannabis-induced disorder
F129	Cannabis use unspecified
F1290	Cannabis use unspecified
F1291	Cannabis use, unspecified, in remission
F1292	Cannabis use, unspecified with intoxication
F12920	cannabis use with intoxication
F12921	cannabis use with intoxication
F12922	cannabis use with intoxication and perceptual disturbance
F12929	cannabis use with intoxication
F1293	Cannabis use with withdrawal
F1295	Cannabis use, unspecified with psychotic disorder
F12950	cannabis use with delusions
F12951	Cannabis use with hallucinations
F1298	Cannabis use, cannabis induced disorder
F12959	cannabis use with psychotic disorder
F12980	Cannabis use, unspecified with anxiety disorder
F12988	cannabis use
F1299	Cannabis use with cannabis induced disorder, unspecified

© 2023 Raffa BJ et al. *JAMA Network Open.*

F149	Cocaine use unspecified
F1490	Cocaine use, unspecified, uncomplicated
F1492	Cocaine use intoxication
F1491	Cocaine use, unspecified, in remission
F14920	Cocaine use, unspecified with intoxication, uncomplicated
F14921	Cocaine use intoxication
F14922	Cocaine use intoxication
F14929	Cocaine use unspecified with intoxication
F1493	Cocaine use, unspecified with withdrawal
F1494	Cocaine use, unspecified with cocaine-induced mood disorder
F14950	Cocaine use, unspecified with cocaine-induced psychotic disorder with delusions
F14951	Cocaine use, unspecified with cocaine-induced psychotic disorder with hallucinations
F14959	Cocaine use, unspecified with cocaine-induced psychotic disorder, unspecified
F14980	Cocaine use, unspecified with cocaine-induced anxiety disorder
F14981	Cocaine use, unspecified with cocaine-induced sexual dysfunction
F14982	Cocaine use, unspecified with cocaine-induced sleep disorder
F14988	Cocaine use, unspecified with other cocaine-induced disorder
F1499	Cocaine use, unspecified with unspecified cocaine-induced disorder
F15921	Poisoning by Amphetamines
F15929	Poisoning by Amphetamines
F1593	Poisoning by Amphetamines
R780	Finding of alcohol in blood
R782	Finding of cocaine in blood
T400X1	Poisoning by opium
T400X1A	Poisoning by opium
T400X1D	Poisoning by opium
T400X1S	Poisoning by opium
T400X3A	Poisoning by opium
T400X3	Poisoning by opium
T400X3D	Poisoning by opium
T400X3S	Poisoning by opium
T400X4A	Poisoning by opium
T400X4	Poisoning by opium
T400X4D	Poisoning by opium
T400X4S	Poisoning by opium
T401	Poisoning by and adverse effect of heroin
T401X1	Poisoning by heroin, accidental
T401X1A	Poisoning by heroin, accidental-initial encounter

© 2023 Raffa BJ et al. *JAMA Network Open.*

T401X1D	Poisoning by heroin, accidental-subsequent encounter
T401X1S	Poisoning by heroin, accidental-sequela
T401X2A	Poisoning by heroin, intentional self-harm, initial encounter
T401X2D	Poisoning by heroin, intentional self-harm, subsequent encounter
T401X2S	Poisoning by heroin, intentional self-harm, sequela
T401X3	Poisoning by heroin, assault
T401X3A	Poisoning by heroin, assault-initial encounter
T401X3D	Poisoning by heroin, assault-subsequent encounter
T401X3S	Poisoning by heroin, assault-sequela
T401X4	Poisoning by heroin, undetermined
T401X4A	Poisoning by heroin, undetermined-initial encounter
T401X4D	Poisoning by heroin, undetermined-subsequent encounter
T401X4S	Poisoning by heroin, undetermined, sequela
T402X	Poisoning by other opioids
T402X1	Poisoning by other opioids
T402	Poisoning by other opioids
T402X1A	Poisoning by other opioids
T402X1D	Poisoning by other opioids
T402X1S	Poisoning by other opioids
T402X3	Poisoning by other opioids
T402X3A	Poisoning by other opioids
T402X3D	Poisoning by other opioids
T402X3S	Poisoning by other opioids
T402X4	Poisoning by other opioids
T402X4A	Poisoning by other opioids
T402X4D	Poisoning by other opioids
T402X4S	Poisoning by other opioids
T403X1A	Poisoning by methadone, accidental (unintentional), initial encounter
T403X1D	Poisoning by methadone, accidental (unintentional), subsequent encounter
T403X1S	Poisoning by methadone-accidental-sequela
T403X4S	Poisoning by methadone-undetermined-Sequela
T403X3A	Poisoning by methadone-assault-IE
T403X4	Poisoning by methadone-undetermined
T403X3D	Poisoning by methadone-assault-SE
T403X1D	Poisoning by methadone-accidental-SE
T403X3	Poisoning by methadone-assault
T403X3S	Poisoning by methadone-assault-sequela
T40414S	Poisoning by fentanyl or fentanyl analogs-undetermined, Sequela

© 2023 Raffa BJ et al. *JAMA Network Open.*

T403X1A	Poisoning by methadone-accidental-IE
T403X1	Poisoning by methadone-accidental
T403X4A	Poisoning by methadone, undetermined IE
T40414D	Poisoning by fentanyl or fentanyl analogs-undetermined, SE
T40414A	Poisoning by fentanyl or fentanyl analogs-undetermined, IE
T40413D	Poisoning by fentanyl or fentanyl assault-SE
T40413A	Poisoning by fentanyl or fentanyl assault-IE
T403X4D	Poisoning by methadone, undetermined SE
T40411S	Poisoning by fentanyl or fentanyl analogs-accidental-sequela
T40416D	Poisoning by fentanyl or fentanyl analogs-accidental-subsequent encounter
T40416A	Poisoning by fentanyl or fentanyl analogs-accidental-initial encounter
T40411	Poisoning by fentanyl or fentanyl analogs-accidental
T4041	Poisoning by fentanyl or fentanyl analogs
T40411D	Poisoning by fentanyl or fentanyl analogs-accidental SE
T40411A	Poisoning by fentanyl or fentanyl analogs-accidental IE
T40413S	Poisoning by fentanyl -assault-sequela
T40413	Poisoning by fentanyl -assault
T40491	Poisoning by synthetic narcotics
T40494A	Poisoning by synthetic narcotics
T40494	Poisoning by synthetic narcotics
T40494S	Poisoning by synthetic narcotics
T40494D	Poisoning by synthetic narcotics
T40491A	Poisoning by synthetic narcotics
T40491D	Poisoning by synthetic narcotics
T40491S	Poisoning by synthetic narcotics
T40493A	Poisoning by synthetic narcotics
T40493D	Poisoning by synthetic narcotics
T40493S	Poisoning by other synthetic narcotics, assault, sequela
T405X	Poisoning by cocaine
T405X1	poisoning by cocaine
T405X1A	Poisoning by cocaine, accidental (unintentional), initial encounter
T405X1D	Poisoning by cocaine, accidental (unintentional), subsequent encounter
T405X1S	Poisoning by cocaine, accidental
T405X2A	Poisoning by cocaine, intentional self-harm, initial encounter
T405X2D	Poisoning by cocaine, intentional self-harm, subsequent encounter
T405X2S	Poisoning by cocaine, intentional self-harm, sequela
T405X3	Poisoning by cocaine, assault
T405X3A	Poisoning by cocaine, Assault-initial encounter

© 2023 Raffa BJ et al. *JAMA Network Open.*

T405X3D	Poisoning by cocaine, assault, subsequent encounter
T405X3S	Poisoning by cocaine-assault sequela
T405X4	Poisoning by cocaine, undetermined
T405X4A	Poisoning by cocaine-initial encounter
T405X4D	poisoning by cocaine-subsequent encounter
T405X4S	Adverse effect of cocaine, sequela
T405X5A	Adverse effect of cocaine, initial encounter
T405X5	Adverse effect of cocaine
T405X5D	Adverse effect of cocaine, subsequent encounter
T405X5S	Adverse effect of cocaine, sequela
T40601A	Poisoning by unspecified narcotics, accidental (unintentional), initial encounter
T40601	Poisoning by other narcotics
T40601D	Poisoning by unspecified narcotics, accidental (unintentional), subsequent encounter
T40601S	Poisoning by unspecified narcotics, accidental (unintentional), sequela
T40603A	Poisoning by other narcotics
T40603D	Poisoning by other narcotics
T40603S	Poisoning by unspecified narcotics, assault, sequela
T40604A	Poisoning by other narcotics
T40604D	Poisoning by other narcotics
T40601D	Poisoning by other narcotics
T40601A	Poisoning by other narcotics
T40601S	Poisoning by other narcotics
T40691	Poisoning by other narcotics
T40604S	Poisoning by unspecified narcotics, undetermined, sequela
T40691A	Poisoning by other narcotics
T40691D	Poisoning by other narcotics
T40691S	Poisoning by other narcotics
T40694A	Poisoning by other narcotics
T40694D	Poisoning by other narcotics
T40694S	Poisoning by other narcotics, undetermined, sequela
T407	Poisoning by or adverse effect due to use cannabis
T40711A	Poisoning by cannabis, accidental (unintentional), initial encounter
T40711D	Poisoning by cannabis, accidental (unintentional), subsequent encounter
T40711S	Poisoning by cannabis, accidental (unintentional), sequela
T40712A	Poisoning by cannabis, intentional self-harm, initial encounter
T40712D	Poisoning by cannabis, intentional self-harm, subsequent encounter
T40712S	Poisoning by cannabis, intentional self-harm, sequela
T40713A	Poisoning by cannabis, assault, initial encounter

© 2023 Raffa BJ et al. *JAMA Network Open.*

T40713D	Poisoning by cannabis, assault, subsequent encounter
T40713S	Poisoning by cannabis, assault, sequela
T40714A	Poisoning by cannabis, undetermined, initial encounter
T40714D	Poisoning by cannabis, undetermined, subsequent encounter
T40714S	Poisoning by cannabis, undetermined, sequela
T40715A	Adverse effect of cannabis, initial encounter
T40715D	Adverse effect of cannabis, subsequent encounter
T40715S	Adverse effect of cannabis, sequela
T40721A	Poisoning by synthetic cannabinoids, accidental (unintentional), initial encounter
T40721D	Poisoning by synthetic cannabinoids, accidental (unintentional), subsequent encounter
T40721S	Poisoning by synthetic cannabinoids, accidental (unintentional), sequela
T40722A	Poisoning by synthetic cannabinoids, intentional self-harm, initial encounter
T40722D	Poisoning by synthetic cannabinoids, intentional self-harm, subsequent encounter
T40722S	Poisoning by synthetic cannabinoids, intentional self-harm, sequela
T40723A	Poisoning by synthetic cannabinoids, assault, initial encounter
T40723D	Poisoning by synthetic cannabinoids, assault, subsequent encounter
T40723S	Poisoning by synthetic cannabinoids, assault, sequela
T40724A	Poisoning by synthetic cannabinoids, undetermined, initial encounter
T40724D	Poisoning by synthetic cannabinoids, undetermined, subsequent encounter
T40724S	Poisoning by synthetic cannabinoids, undetermined, sequela
T40725A	Adverse effect of synthetic cannabinoids, initial encounter
T40725D	Adverse effect of synthetic cannabinoids, subsequent encounter
T40725S	Adverse effect of synthetic cannabinoids, sequela
T40726A	Adverse effect of synthetic cannabinoids, sequela
T424X1	poisoning by benzodiazepine
T424X4	poisoning by benzodiazepine
T424X3	poisoning by benzodiazepine
T424X1A	Poisoning by benzodiazepines, accidental (unintentional), initial encounter
T424X1D	Poisoning by benzodiazepines, accidental (unintentional), subsequent encounter
T424X1S	Poisoning by benzodiazepines, accidental (unintentional), sequela
T424X3A	Poisoning by benzodiazepines, assault, initial encounter
T424X3D	Poisoning by benzodiazepines, assault, subsequent encounter
T424X3S	Poisoning by benzodiazepines, assault, sequela
T424X4A	Poisoning by benzodiazepines, undetermined, initial encounter
T424X4D	Poisoning by benzodiazepines, undetermined, subsequent encounter
T4360	poisoning by psychostimulant
T43601	poisoning by psychostimulant

© 2023 Raffa BJ et al. *JAMA Network Open.*

T43603	poisoning by psychostimulant
T436	poisoning by psychostimulant
T424X4S	Poisoning by benzodiazepines, undetermined, sequela
T43601A	Poisoning by unspecified psychostimulants, accidental (unintentional), initial encounter
T43601D	Poisoning by unspecified psychostimulants, accidental (unintentional), subsequent encounter
T43601S	Poisoning by unspecified psychostimulants, accidental (unintentional), sequela
T43602A	Poisoning by unspecified psychostimulants, intentional self-harm, initial encounter
T43602D	Poisoning by unspecified psychostimulants, intentional self-harm, subsequent encounter
T43602S	Poisoning by unspecified psychostimulants, intentional self-harm, sequela
T43603A	Poisoning by unspecified psychostimulants, assault, initial encounter
T43603D	Poisoning by unspecified psychostimulants, assault, subsequent encounter
T43603S	Poisoning by unspecified psychostimulants, assault, sequela
T43604	poisoning by psychostimulant
T43604A	Poisoning by unspecified psychostimulants, undetermined, initial encounter
T43604D	Poisoning by unspecified psychostimulants, undetermined, subsequent encounter
T43604S	Poisoning by unspecified psychostimulants, undetermined, sequela
T43605	poisoning by psychostimulant
T43605A	Adverse effect of unspecified psychostimulants, initial encounter
T43605D	Adverse effect of unspecified psychostimulants, subsequent encounter
T43605S	Adverse effect of unspecified psychostimulants, sequela
T43621A	Poisoning by Amphetamines
T43621D	Poisoning by amphetamines, accidental (unintentional), subsequent encounter
T43621S	Poisoning by amphetamines, accidental (unintentional), sequela
T43622A	Poisoning by amphetamines, intentional self-harm, initial encounter
T43622D	Poisoning by amphetamines, intentional self-harm, subsequent encounter
T4362	Poisoning by Amphetamines
T43622S	Poisoning by amphetamines, intentional self-harm, sequela
T43623A	Poisoning by amphetamines, assault, initial encounter
T43623D	Poisoning by amphetamines, assault, subsequent encounter
T43623S	Poisoning by amphetamines, assault, sequela
T43624A	Poisoning by amphetamines, undetermined, initial encounter
T43624D	Poisoning by amphetamines, undetermined, subsequent encounter
T43624S	Poisoning by amphetamines, undetermined, sequela
T43625A	Poisoning by Amphetamines
T43625D	Adverse effect of amphetamines, subsequent encounter
T43625S	Adverse effect of amphetamines, sequela

© 2023 Raffa BJ et al. *JAMA Network Open.*

T510	Toxic effect of alcohol
T510X	Toxic effect of ethanol
T510X1A	Toxic effect of ethanol, accidental (unintentional), initial encounter
T510X1D	Toxic effect of ethanol, accidental (unintentional), subsequent encounter
T510X1S	Toxic effect of ethanol, accidental (unintentional), sequela
T510X2A	Toxic effect of ethanol, intentional self-harm, initial encounter
T510X2D	Toxic effect of ethanol, intentional self-harm, subsequent encounter
T510X2S	Toxic effect of ethanol, intentional self-harm, sequela
T510X3A	Toxic effect of ethanol, assault, initial encounter
T510X3D	Toxic effect of ethanol, assault, subsequent encounter
T510X3S	Toxic effect of ethanol, assault, sequela
T510X4A	Toxic effect of ethanol, undetermined, initial encounter
T510X4D	Toxic effect of ethanol, undetermined, subsequent encounter
T510X4S	Toxic effect of ethanol, undetermined, sequela
R781	opiate in blood
R780	finding of alcohol in blood

© 2023 Raffa BJ et al. *JAMA Network Open.*
