# Supplementary material for: Ingestion of Illicit Substances by Young Children Before and During the COVID-19 Pandemic
Source: JAMA Netw Open. 2023 Apr 21;6(4):e239549. doi: 10.1001/jamanetworkopen.2023.9549 (PMC10122182; doi:10.1001/jamanetworkopen.2023.9549)
Supplement: Supplement 2. — Data Sharing Statement [file jamanetwopen-e239549-s002.pdf]

Data Sharing Statement

Raffa. Ingestion of Illicit Substances by Young Children Before and During the COVID-19 Pandemic. *JAMA Netw Open*. Published April 21, 2023.
doi:10.1001/jamanetworkopen.2023.9549

Data
Data available: No
